# Estimating the minimal clinically important difference of shoulder functional scores after arthroscopic rotator cuff repair: a prospective study

**DOI:** 10.1007/s00402-024-05222-8

**Published:** 2024-02-22

**Authors:** Aditya A Mukadam, Shalini Nayak, Jaap Willems, Vivek Pandey

**Affiliations:** 1 Department of Orthopedics, Kasturba Medical College, Manipal, Manipal Academy of Higher Education, Udupi, Karnataka 576104 India; 2 Department of Physiotherapy, Manipal College of Health Professions, Manipal Academy of Higher Education, Udupi, Karnataka 576104 India; 3 International Knee and Joint Centre, Abu Dhabi, UAE

**Keywords:** Minimal clinically important difference (MCID), American shoulder and elbow surgeons score, University of California and Los Angeles score, Distribution-based approach, Anchor-based approach, Rotator cuff repair

## Abstract

**Introduction:**

The minimal clinically important difference (MCID) is a valuable tool for patient-based outcome analysis, for which limited data is available in the literature, especially after arthroscopic rotator cuff repair (ARCR). Although several studies have reported MCID after ARCR, few have studied the impact of various clinical factors such as Diabetes, pseudoparalysis, type of cuff repair, and retear over MCID. This study attempts to determine the MCID in shoulder functional scores after ARCR and the impact of various factors on MCID.

**Methods:**

144 patients undergoing ARCR were prospectively evaluated at six and 12 months by ASES and UCLA scores. MCID for American Shoulder and Elbow Surgeons (ASES) and the University of California and Los Angeles (UCLA) scores were calculated using an anchor-based and distribution-based approach. MCID was also calculated for diabetic and non-diabetic patients, smokers vs. non-smokers, presence or absence of pseudoparalysis, type of cuff repair (single row vs. suture bridge), and presence of retears. Uni- and multivariate analysis was performed to identify factors affecting the MCID of both scores.

**Results:**

Mean MCID for ASES score was 13.3 and 16.6 using an anchor-based and distribution-based approach, respectively. For the UCLA score, the mean MCID was 10.0 and 12.6 by anchor-based and 12.6 by distribution-based approach, respectively. Patients with higher pre-operative ASES scores demonstrated lower MCID values. No significant difference was observed in MCID scores of diabetic vs. non-diabetic patients, smoker vs. non-smoker, patients with or without pseudoparalysis, and type of cuff repair. The age, gender, and presence of retear did not affect MCID values.

**Conclusion:**

This study establishes the MCID values of ASES and UCLA scores for rotator cuff repair by anchor and distribution methods. No patient or surgical factors appear to affect the MCID except pre-operative ASES scores.

**Study design:**

Prospective cohort, Level II.

## Introduction

Various techniques used in repairing rotator cuff tear result in comparable clinical outcomes in the long term [1, 2]. One of the standard methods to assess the efficacy of surgical repair is to see whether the reported outcome is statistically significant. However, the data size affects the statistical significance [3]. A large data set can show a statistically significant difference between the outcomes, which may not be clinically meaningful to the patient [3, 4]. Similarly, a clinically meaningful difference is rejected if it fails to achieve statistical difference due to a small data size [5, 6]. Therefore, there has been a gradual shift from relying merely upon statistical significance in patient-reported outcomes to assessing the meaningful clinical impact of observed differences [7, 8].

To bridge this gap between statistically significant value and clinical significance, the concept of minimal clinically important difference (MCID) has been evolving for a long time [9]. The minimal clinically important difference (MCID) of a score is defined as the smallest change in the score, which would signify a clinical improvement in a patient’s symptoms or satisfaction level [10]. MCID can be considered an index for the functional improvement of the patient or how much improvement the patient needs to observe a positive change in their activities of daily living [11]. MCID also directly addresses the limitations of assessing statistical significance in isolation, particularly the possibility that studies may find statistical significance that does not have any clinical importance to patients and clinicians [12].

Although many methods exist to calculate MCID, two commonly deployed methods are anchor-based and distribution methods. The anchor-based approach utilizes a specific anchor question [13]. In contrast, the distribution-based approach is based on the patient’s pre-operative characteristics and thus needs careful sample selection representing the general population [14]. Different studies have shown a uniform pattern of MCID values by the anchor-based approach compared to the distribution-based approach, which is clinically relevant and statistically significant [12, 14–16].

Recently, much work has been done to evaluate MCID for shoulder scores, such as the Constant Murley, American Shoulder and elbow surgeons (ASES), and the University of California and Los Angeles (UCLA) score [17–20]. Nevertheless, a systematic review by Jones et al. stressed the variable methodology and paucity of adequate data and reporting on MCID values regarding shoulder scores in the literature [21]. Currently, MCID values are taken into account while calculating the sample size for a study [22], and therefore, it is essential to know the reasonable range of MCID after rotator cuff repair.

Furthermore, except for a few [17, 23], most studies assessing MCID have not considered other patient factors such as Diabetes mellitus, smoking, pseudoparalysis, type of cuff repair (single row vs. suture bridge technique), and retears while reporting MCID for shoulder scores. The effects of these factors are well established after rotator cuff repair [17, 24–27]; therefore, their impact on MCID needs to be probed in detail.

In this prospective study, we have attempted to determine MCID in ASES and UCLA shoulder functional scores and the effect of various factors on MCID.

## Materials and methods

This is a prospective study from 2019 to 2021 of patients who underwent ARCR at our institute. After approval by the institutional ethics committee (IEC/613/2019) and Clinical Trials Registry-India (CTRI), patients were evaluated considering the inclusion and exclusion criteria of the study.

### Sample size calculation

Considering a minimum of 10% prevalence of full-thickness rotator cuff tear in the population, a minimum sample size of 139 patients was needed for the study with 95% confidence intervals with a 5% margin of error.

### Inclusion and exclusion criteria

All patients in the age group of 40–75 years, clinically and radiologically diagnosed with a complete rotator cuff tear undergoing arthroscopic repair for the same by a single surgeon, were included in this study. Preoperative pseudoparalysis was noted in patients, which was defined as a lack of active elevation of the shoulder less than 90^0^ without any neurological injury [28]. A history of Diabetes and smoking was also noted. Exclusion criteria included previously workers’ compensation patients, operated ipsilateral shoulder, isolated subscapularis repair, irreparable rotator cuff tears or partial repairs, associated frozen shoulder which required intraoperative release, pre-existing degenerative glenohumeral arthritis (Samilson-Prieto grade 2 and above) [29], or inflammatory arthritis. ASES and UCLA were noted preoperatively.

### Surgical technique

All patients were operated on by a single senior surgeon. The surgery was performed in a sloppy lateral position under general anaesthesia and interscalene block. Diagnostic arthroscopy was performed, and intra-op findings were recorded. An adequate bursectomy was performed for adequate visualization of the torn cuff. Acromioplasty was added only if there was an acromial spur. Biceps tenotomy or biceps tenodesis was performed based on the condition of the biceps, age, and functional demands of the patient. The footprint was prepared in a standard fashion using a radiofrequency device and motorized burr. The edges of the torn cuff were then pulled laterally to check the adequacy of the reduction of the cuff over the greater tuberosity. Standard releases were performed in case of a retracted cuff. The type of cuff repair [single or double-row suture bridge (transosseous equivalent)] was performed based on the size of the tear (Cofield classification) [30], retraction of tendons, delamination characteristics, and reducibility of the tear. Generally, small to medium-sized tears were repaired using a single-row (SR) technique, whereas large to massive tears were managed using a double-row suture bridge (DRSB) technique.

### Rehabilitation

All patients were managed with an arm sling post-surgery for 4–6 weeks. Small to medium-sized tears were allowed gentle passive mobilization in the immediate post-operative period, whereas large-massive tears remained immobilized for six weeks. Active assisted mobilization was initiated after six weeks, followed by active mobilization after ten weeks. Rotator cuff strengthening was initiated after 12 weeks. Return to regular activities and sports were allowed after six to nine months, depending upon functional improvement and strength.

### Shoulder functional outcome measures

All patients received shoulder-specific questionnaires: American Shoulder and Elbow Surgeons (ASES) standardized shoulder assessment form and the University of California and Los Angeles score [31, 32]. ASES score is a 100-point scale that evaluates two dimensions of shoulder function: pain and performance in activities of daily living, whose psychometric properties, validity, reliability, and responsiveness have been well-established in assessing shoulder conditions [33]. UCLA score is a 35-point scale comprised of subjective (pain, function, and satisfaction) and objective (range of motion, strength) components. UCLA’s reliability, validity, and responsiveness are inferior to ASES [33].

### Rotator cuff repair structural assessment

All patients underwent ultrasonographic evaluation of the repaired rotator cuff at 12 weeks and the end of one year to assess tendon integrity and any retears. The ultrasound report was broadly classified into three categories. Type I, normal thickness with homogeneously hyperechoic tendon or partial hypoechogenicity or heterogenicity or insufficient thickness without discontinuity indicating ‘complete healing’; Type II, the presence of a minor discontinuity or a focal partial defect indicating ‘partial tear’; and Type III, the presence of a significant discontinuity or a ‘full-thickness tear.’ Gartsman et al. and Gwark et al. used similar criteria for ultrasound assessment of the post-operative healing status of the cuff [34, 35].

### MCID of ASES and UCLA determination methods

Two methods, anchor and distribution, were deployed to calculate the MCID of ASES and UCLA. The anchor-based approach utilizes a specific anchor question. In contrast, the distribution-based approach is based on the patient’s pre-operative characteristics and thus needs careful sample selection representing the general population.

1. Anchor-based approach: The anchor in this study was the ‘satisfaction anchor,’ whether the patient was clinically (subjectively) satisfied at the follow-up timeline of 6 months and 12 months or not. The responses were prospectively recorded in binary form as ‘yes’ if the patient was ‘satisfied and better’ or ‘no’ if they were ‘dissatisfied and worse.’ The mean difference was calculated for both scoring systems (ASES and ULCA) and analyzed by paired t-test.
2. Distribution-based approach: This method relies on the baseline statistical values of the sample and denotes change considering these baseline characteristics. MCID for both scores was calculated using standard deviation (SD), standard error of the mean (SEM), and effect size (z). Mean change and standard deviation values for a 95% confidence interval have been used for analysis.

Anchor-based analysis is more patient-oriented and better representative of functional improvement in patients’ status [13]. In contrast, the distribution-based approach is based on statistical methods; therefore, it defies the essence of MCID to focus on clinical improvement rather than statistical significance [9, 36]. Different studies have shown a uniform pattern of MCID values by the anchor-based approach compared to the distribution-based approach, which is clinically relevant and statistically significant [12, 14–16].

### Statistical analysis

The statistical analysis was performed using SPSS 22.0 software (IBM, NY, USA). The data were presented as the mean and standard deviation for continuous variables and percentages for the categorical variables. Paired t-test was used to compare the paired means. The MCID was calculated for ASES and UCLA scores using the anchor-based approach with ROC (receiver operating characteristic) analysis. The area under curve (AUC) of 0.7 to 0.8 is considered acceptable, 0.8 to 0.9 is considered excellent, and more than 0.9 is considered outstanding [37]. MCID was also calculated by the distribution-based approach using the mean change, standard error of the mean, standard deviation, effect size, and paired t-statistics. The *p*-value of < 0.05 was considered significant. The data was also analyzed by dividing pre-operative scores into different ranges and calculating MCID scores for different ranges of pre-operative scores.

## Results

This prospective study analyzed 144 patients comprising 93 males (64.6%) and 51 females (35.4%) to assess MCID values of ASES and UCLA scores. The baseline characteristics of patients are mentioned in Table 1. All patients were followed up for one year, and no patient was lost to follow-ups. There was a statistically significant increase in mean ASES and UCLA scores postoperatively compared to pre-operative scores (Table 2). We had a total of 5 retears; one full thickness in the posterior third of the supraspinatus tendon (6 mm), and the other four were partial discontinuity in the tendon.

**Fig. 1:**
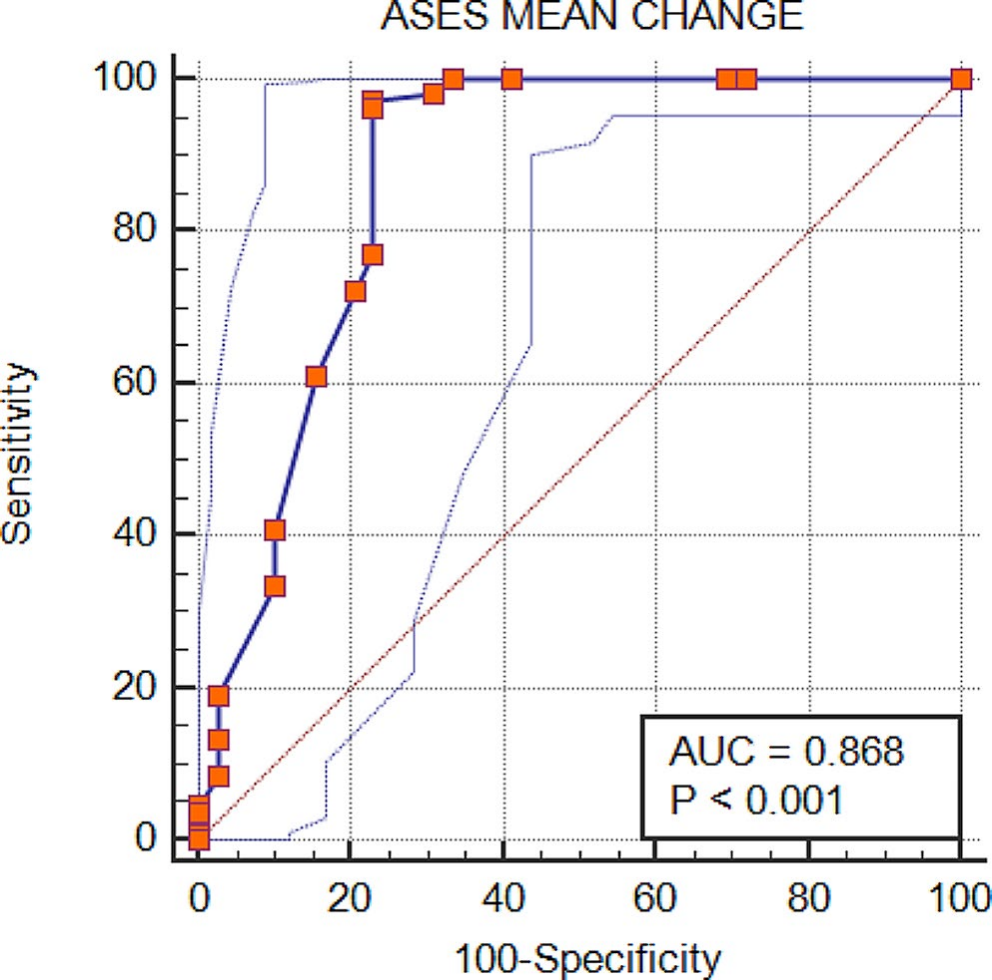
Receiver operating curve showing area under curve for ASES score. ASES: American shoulder and elbow surgeon

**Fig. 2:**
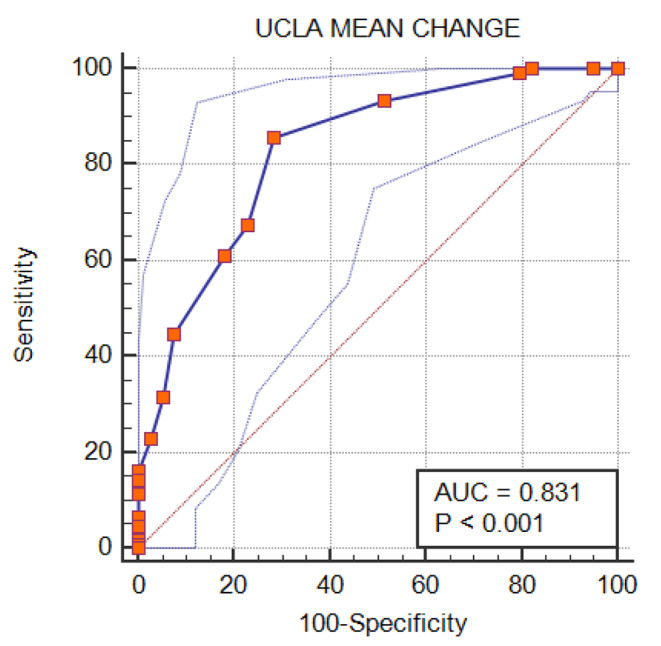
Receiver operating curve showing area under curve for UCLA score. UCLA: university of California and Los Angeles

*MCID of scores using anchor method-* Based upon ROC and AUC, an MCID cutoff value of 13.3 was found for ASES, representing 86.8% AUC (Fig. 1). Similarly, an MCID cutoff value of 10.0 was found for the UCLA score, representing 83.1% AUC (Fig. 2).

The AUC for ASES and UCLA is above 0.8 and, therefore, considered excellent (Table 3).

### MCID of scores using distribution method

For the ASES score and UCLA scores, MCID of 16.6 (8.0–25.2) and MCID of 12.6 (5.7–19.5) corresponded to 95% confidence intervals, respectively.

Patients were assessed on follow-up regarding their subjective improvement of symptoms and functional activities and clinically examined for shoulder movements and power. A total of 111 patients demonstrated a change of ASES score change > 13.3 points, of which 102 (91.9%) were found to be satisfied, while the remaining nine patients (8.1%) were not satisfied (*p* < 0.001) [Table 4]. Out of the 101 patients with a UCLA score change of > 10 points, 90 patients (89.1%) demonstrated satisfaction postoperatively, while 11 patients (10.9%) did not (*p* < 0.001) [Table 4].

The data were also analyzed by dividing pre-operative PROM scores into different categories. ASES score was divided into three categories: equal to or less than 30 points, 31–50 points, and more than 50 points for the ASES score. It was noted that MCID scores with 95% confidence intervals were 19.04 (± 4.9), 16.52 (± 4.3), and 13.89 (± 1.9), respectively. Similar groups of equal to or less than 10, 11–20, and more than 20 points for UCLA score showed MCID values of 17.9 (± 3.4), 11.84 (± 2.6), and 9 (± 1.4), respectively. Thus, higher pre-operative scores had lower MCID values for ASES and UCLA scores.

Furthermore, considering age, gender, preoperative ASES and ULCA scores, Diabetes, smoking, pseudoparalysis, type of repair, and retears, the results of both uni- and multivariate logistic analyses indicated that the only variable significantly influencing the MCID is the preoperative ASES score (Tables 5 and 6).

**Table 1:** Demographic and baseline descriptive data of 144 patients. DSRB: double row suture bridge; Type of subscapularis tear is based upon Lafosse classification. Note that Supraspinatus was torn in all cases, while partial and complete infraspinatus tears are mentioned separately

Mean age ± SD (in years)		55.62 ± 9.22
Gender	Male	93 (64.6%)
	Female	51 (35.4%)
Side of involvement	Right	106 (73.61%)
	Left	38 (26.39%)
Presence of Diabetes	Yes	33 (22.91%)
	No	111 (77.08%)
Thyroid disorder	Yes	11 (7.63%)
	No	133 (92.36%)
Smoking	Yes	45 (31.2%)
	No	99 (68.75%)
Pseudopalsy	Present	61 (42.36%)
	Absent	83 (57.63%)
Full-thickness supraspinatus tear		144 (100%)
Partial thickness infraspinatus tear		37 (25.7%)
Full-thickness infraspinatus tear		22 (15.3%)
Subscapularis tear (33)	Type 1	5 (3.47%)
	Type 2	11 (7.63%)
	Type 3	13 (9.02%)
	Type 4	4 (2.77%)
Supraspinatus fatty infiltration (n = 144)	Grade 0–1	97 (67.36%)
	Grade 2	40 (27.7%)
	Grade 3	7 (1.92%)
Infraspinatus fatty infiltration (n = 59)	Grade 0–1	39 (66.1%)
	Grade 2	17 (28.8%)
	Grade 3	3 (5.08%)
Subscapularis fatty infiltration (n = 33)	Grade 0–1	25 (75.7%)
	Grade 2	5 (15.1%)
	Grade 3	3 (9.1%)
Type of Cuff repair	Single row	48 (33.3%)
	DRSB	96 (66.6%)
Biceps procedure	None	78 (54.1%)
	Tenotomy	54 (37.5%)
	Tenodesis	12 (8.33%)
Acromioplasty	Yes	13 (9.9%)
	No	131 (90.1%)
Retear	Yes	5 retears. 4 partial; 1 full thickness
	No	139

**Table 2:** ASES and UCLA scores at preoperative, six, and twelve months postoperatively. SD: standard deviation; ASES: American shoulder and elbow score; UCLA: University of California and Los Angeles; Preop: preoperative

	Score timeline	Mean (range)	SD	Mean difference ± SD (Preop. vs. six months)	Mean ASES difference ± SD (Preop. vs. 12 months)
ASES score	Preoperative	39.4 (26.6–58.3)	5.8	Mean difference = 9.05 +/-3.5 *p* < 0.001	Mean difference= 16.59+/-4.4 *p* < 0.001
	6 months post-operative	48.5 (35.0-66.6)	6.2		
	12 months post-operative	56.1 (41.6–70.0)	6.2		
UCLA score	Preoperative	15.1 (6.0–23.0)	4.1	Mean difference = 7.16 +/-4.11. *p* < 0.001	Mean difference = 12.60 +/-3.49. *p* < 0.001
	6 months post-operative	22.3 (9.0–31.0)	4.8		
	12months post-operative	27.7 (19.0–32.0)	2.9		

**Table 3:** MCID cutoff for ASES and UCLA scores. AUC: area under curve; PPV: positive predictive value; NPV: negative predictive value

Scores	MCID Cutoff	AUC	Sensitivity	Specificity	PPV	NPV
ASES	> 13.3	0.86	97.1	76.9	91.9	90.9
UCLA	> 10	0.83	85.7	71.8	89.1	65.1

**Table 4:** Comparison of patient satisfaction with MCID values in ASES and UCLA scores. ASES: American shoulder and elbow surgeons; UCLA: University of California and Los Angeles

Score	MCID by Anchor method	Patient’s satisfaction		*p*-value
		Yes	No	< 0.001
ASES	> 13.3	102 (91.9%)	9 (8.1%)	
	< 13.3	3 (9.1%)	30 (90.9%)	
UCLA	> 10	90 (89.1%)	11 (10.9%)	< 0.001
	< 10	15 (34.9%)	28 (64.1%)	

**Table 5:** Logistic regression analysis of factors affecting MCID in ASES score. Numbers highlighted in bold signify statistical significance. ASES: American shoulder and elbow surgeons; DRSB: double row suture bridge

Variable	Univariate analysis (*p*-value)	Multivariate analysis (*p*-value)	95% Confidence interval
*Pre-operative ASES score*	**0.036**	**0.020**	1.01, 1.20
*Age*	0.310	0.169	0.92, 1.01
*Gender*	0.514	0.691	0.32, 2.13
*Diabetes*	0.967	0.592	0.315, 1.93
*Pseudopalsy*	0.226	0.223	0.70, 4.39
*Smoking*	0.371	0.592	0.28, 2.04
*DRSB repair*	**0.022**	0.394	0.63, 3.17
*Retears*	0.452	0.632	0.247, 1.82

**Table 6:** Logistic regression analysis of variables associated with achieving MCID in UCLA score. UCLA: University of California and Los Angeles: DRSB: double row suture bridge

Variable	Univariate analysis- (*p*-value)	Multivariate analysis (*p*-value)	95% confidence interval
*Preoperative UCLA score*	0.193	0.067	0.99, 1.29
*Age*	0.222	0.110	0.91, 1.00
*Gender*	0.785	0.723	0.33, 2.16
*Diabetes*	0.516	0.559	0.30, 1.88
*Pseudopalsy*	0.851	0.195	0.68, 6.30
*Smoking*	0.279	0.479	0.26, 1.87
*DRSB repair*	0.188	0.479	0.60, 2.95
*Retear*	0.258	0.864	0.11, 2.81

## Discussion

This study establishes the MCID values for ASES and UCLA scores using anchor and distribution methods in patients undergoing rotator cuff repair. The results indicate that patients who had higher preoperative ASES scores had lower MCID values. However, no discernible factors were found to have an impact on MCID values of the UCLA scoring system. Furthermore, our study found no effect of age, gender, Diabetes, smoking, pseudoparalysis, repair technique opted and retears over the MCID of either score.

Although many studies have recently reported varying ASES and UCLA MCID after rotator cuff repair, the variable methodology adopted in these studies has resulted in varying values of MCID (Table 7).

**Table 7:** Synopsis of various studies reporting MCID values of ASES and UCLA scores in patients following rotator cuff repair. SR: single row; DRSB: double row suture bridge; ASES: American Shoulder And Elbow Score; UCLA: University of California and Los Angeles; MCID: Minimal clinically important difference

Author, Year	Type of study, patients, and follow-up	Characteristics of study	MCID estimation method	ASES	UCLA
*Present (our) study*	Prospective, 144 patients, 12 months	-Only complete tears and complete repairs are included -Both SR and DRSB repair	*Anchor* (2 questions on satisfaction) and *Distribution*	13.3 (anchor) 16.6 (distribution)	10 (anchor) 12.6 (distribution)
Malavota et al. 2022 [18]	Retrospective, 289 shoulders, 12 months	-SR repair - Only 88.6% were complete repair	*Anchor* (2 questions on satisfaction) and *Distribution*	6.1 (Anchor) 10.5 (Distribution)	2.5 (Anchor) 4.5 (Distribution)
Sheng Xu et al. 2020 [38]	Retrospective, 306 patients, 24 months	-Both Partial and complete SS tear included - No details on the type of repair	Anchor (patient satisfaction- 6 point scale and expectation fulfillment- 7 point scale)	-	3
Kim et al. 2020 [23]	Prospective, 82 patients, 12 months	-Both Partial and complete tears included - Both SR and DRSB repair	*Anchor* (4-question scale)	21	6
Tashjian et al. 2020 [19]	Retrospective, 202 patient, 12 months	-Only complete tears included -Both SR and DR repairs	*Anchor* (4-question scale)	27.1	-
Cvetanovich et al. 2019 [17]	Retrospective, 288 patients, 12 months	-No details on the type of tears included (partial or complete) - Both SR and DRSB repairs	*Anchor* (satisfaction- two questions; pain- 15-point global scale)	11.1	-

The MCID values for ASES (13.3) using an anchor-based approach in our study were similar to one reported by Cvetanovich et al. (11.1) and the systematic review by Jones et al. (15.5) [17, 21]. However, the ASES MCID values reported by Kim et al. (21) and Tashjian et al. (27.1) are higher than our values [19, 23], whereas Malavolta et al. reported lower ASES MCID of 6.1 and 10.5 by anchor and distribution methods, respectively [18]. The difference between ours and Kim et al. and Tashjian et al. can be explained by the fact that these authors have used 4-item questions to assess MCID, whereas ours used a binary method which is similar to the one used by Malavolta et al. Furthermore, the difference between Malavolta et al. and ours could be explained possibly by the study design (retrospective), only single row type repair, and the inclusion of 11.4% of incomplete rotator cuff repairs [18]. The MCID value of UCLA in our study was higher (10) than the ones reported by other authors, which ranged from 2.5 to 6 [18, 23, 38].

Another important observation was made in our study that considering MCID 13.3 and 10 as cutoff (by anchor method) for ASES and UCLA, respectively, almost 90% of patients in both groups were satisfied after achieving this MCID value (Table 4, *p* < 0.001), implying that these two values (13.3 and 10) can be considered as a significant cutoff to study patients undergoing arthroscopic rotator cuff repair for a full thickness tear.

MCID for ASES and UCLA continues to vary in different studies due to the diverse population of the patient and their expectations, baseline characteristics, varying methodology (retrospective/prospective), the spectrum of disease (rotator cuff tendinopathy and tears-partial or complete) studied, lack of data and type of management (conservative or operative, type of repair) offered [21, 22, 39]. Therefore, more studies from specific regions with defined populations and patient characteristics would help establish a relatively narrow range of MCID, which will help clinicians assess patient clinical performance in their practice.

### Effect of preoperative scores on MCID

Patients with higher preoperative ASES scores achieving MCID earlier indicate that patients with a lesser preoperative functional deficit would normalize earlier than those with a higher functional deficit. Cvetanovich et al. reported a similar finding [17]. Regarding the UCLA score, we did not find an influence of any preoperative factor over MCID. In contrast, Malavolta et al. reported that patients with lower UCLA scores had higher MCID and vice versa [18]. The difference could be due to different population characteristics.

### Role of age, gender, diabetes, smoking, and pseudoparalysis

Although ours and other studies failed to confirm the role of age in MCID, Malavolta et al. reported that age is a confounding factor in the MCID of ASES and UCLA scores [18]. However, the effect of age on MCID is less profound than preoperative scores [18]. Gender and Diabetes do not seem to affect the MCID of either score, which is similar to that of other studies [17, 18]. Concerning smoking, contrasting results have been reported by two studies. In contrast to Cvetanovich et al., who reported that smoking influences the MCID of ASES, Kim et al. did not observe this effect [17, 23]. . Even our study found no difference between smokers and non-smokers. Concerning the impact of pseudoparalysis, our study has not identified any significant effect on the MCID of either score. In addition, no other study has attempted to validate this parameter.

### Type of cuff repair

Regarding the type of cuff repair affecting MCID, the study by Cvetanovich et al. concluded that single-row repair could fail to achieve a clinically significant outcome [17]. In contrast, ours and Kim et al. did not find any effect of type of repair over MCID. However, Cvetanovich et al. state that single-row repair resulting in inferior clinical outcomes might have resulted from their retrospective study design and potential confounding variables [17].

### Strength of our study

The main strength of our study is that it is a prospective study with uniform preoperative evaluation of patients with full-thickness rotator cuff tears. Many other major studies in the literature calculating MCID after rotator cuff repair are retrospective [17–19, 38], which can have a recall bias. Further, patients undergoing only arthroscopic rotator cuff repair, which was completely reparable, were included in this study, increasing the data’s validity due to the exclusion of non-operatively managed, partial repair, and open cuff repair cases [40, 41]. Furthermore, the data in our study is analyzed using an anchor and a distribution-based approach, which adds to the statistical correlation of assessment of preoperative and postoperative functional scores to estimate MCID values.

### Limitation of the study

Our study has several limitations. The patients were followed up for one year postoperatively; a longer follow-up may change results. However, a study by Xu et al. found no significant change in MCID scores between 12 and 24 months [38]. Another limitation of this study is that the sample has not evaluated the association of confounding variables such as the tear size, associated comorbidities body mass index with attaining MCID, and worker compensation group. Further, intraoperative factors such as biceps tenotomy vs. tenodesis and bony acromioplasty, which was decided based on arthroscopy findings, may affect the outcome but were not included in this study. However, such factors vary according to the individual patient and are primarily challenging to control.

## Conclusion

This study reliably concludes MCID values for ASES and UCLA scores after arthroscopic rotator cuff repair based upon anchor and distribution-based approach. In addition, apart from the preoperative ASES score, no other preoperative factors appear to be influencing the MCID. The findings of this study will help to counsel the patients regarding the timing and scale of clinical improvement after the cuff repair and their return to activities of daily living. It will also help determine the sample size for various prospective rotator cuff repair trials. Furthermore, we recommend that more prospective studies with a larger sample size are required to confirm or refute the roles of various preoperative factors affecting MCID.

## Declarations

### Institution where the study was performed

Kasturba Medical College, Manipal

Manipal Academy of Higher Education, Karnataka, India-576104

### CTRI approval

REF/2019/11/022145
